# NEPE Propellant Mesoscopic Modeling and Damage Mechanism Study Based on Inversion Algorithm

**DOI:** 10.3390/ma17061289

**Published:** 2024-03-11

**Authors:** Zhenyuan Hu, Kaining Zhang, Qiqi Liu, Chunguang Wang

**Affiliations:** State Key Laboratory for Strength and Vibration of Mechanical Structure, School of Aerospace, Xi’an Jiaotong University, Xi’an 710049, China; 3122106109@stu.xjtu.edu.cn (Z.H.); knzhang@stu.xjtu.edu.cn (K.Z.); qiqiliuxjtu@163.com (Q.L.)

**Keywords:** NEPE propellant, RVE model, CZM, inversion algorithm, damage evolution process

## Abstract

To accurately characterize the mesoscopic properties of NEPE (Nitrate Ester Plasticized Polyether) propellant, the mechanical contraction method was used to construct a representative volume element (RVE) model. Based on this model, the macroscopic mechanical response of NEPE propellant at a strain rate of 0.0047575 s^−1^ was simulated and calculated, and the parameters of the cohesive zone model (CZM) were inversely optimized using the Hooke–Jeeves algorithm by comparing the simulation results with the results of the uniaxial tensile test of NEPE propellants. Additionally, the macroscopic mechanical behavior of NEPE composite solid propellants at strain rates of 0.00023776 s^−1^ and 0.023776 s^−1^ was also predicted. The mesoscopic damage evolution process of NEPE propellants was investigated by the established model. The study results indicate that the predicted curves are relatively consistent with the basic features and change trends of the test curves. Therefore, the established model can effectively simulate the mesoscopic damage process of NEPE composite solid propellants and their macroscopic mechanical properties.

## 1. Introduction

Composite solid propellant is a viscoelastic particle-reinforced material, typically including an oxidizer, metal powder, and binder [1]. The overall mechanical characteristics of composite solid propellants are intricately linked to the internal mesoscopic of the propellant. Consequently, investigating the mechanism of damage and destruction in the propellant, and interpreting its macroscopic mechanical behavior from a mesoscopic perspective, is a valuable approach to inspire the development of propellants with enhanced performance.

In the macroscopic view, the phenomenological method is mostly used to introduce the phenomenological constants and functions of deformation characteristics from the perspective of continuum mechanics to construct the nonlinear mesoscopic damage constitutive of the propellant. Based on the perspective of thermodynamics and energy, Schapery [2] proposed a very typical nonlinear viscoelastic constitutive model when studying the particle-reinforced composite constitutive model. The soften function and damage internal variables based on the online viscoelastic constitutive model were introduced to characterize the nonlinearity of the propellant damage. After that, based on the time–temperature superposition principle, Park [3] proposed a constitutive model on the growing damage behavior of particulate composites with changing microstructures. Moreover, in order to describe the mechanical response of propellants under complex loads, scholars have established various nonlinear constitutive models. In high-strain-rate impact loading conditions, Sook-Ying Ho [4] presented a constitutive model for composite solid propellants by combining damage mechanics and nonlinear viscoelasticity. To describe the stress–strain response of high-elongation solid propellants, Swanson and Christensen [5] proposed a nonlinear constitutive model including a time correlation function and a strain-softening function. Macroscopic methodologies fulfill the requirement for examining the mechanical properties of propellants. However, the progression of damage in the mesoscopic structure has been somewhat disregarded.

In terms of meso-research, the study of composite propellant can be approached through two methods: testing and simulation. Testing involves observing the changes in the propellant’s mesoscopic structure using CT [6] and SEM [7], while numerical simulation technology simulates the evolution process of fine damage in the propellant. In propellant mesoscopic experimental studies. Deng et al. [8] investigated the mechanical properties of the propellant at lower strain rates by means of long-term relaxation tests and creep tests. And the mesoscopic structural features of the propellant surface were analyzed by the SEM technique. Hwang et al. [9] conducted low-velocity shock tests to investigate the dynamic properties of solid propellants under different operating temperature conditions. Sun et al. [10] conducted impact tests on NEPE propellants using split Hopkinson rods to investigate the mechanical properties of the propellant at high strain rates. And the mesoscopic structure of the propellant was observed by SEM. In propellant mesoscopic simulation studies. Tan et al. [11,12,13] proposed a nonlinear cohesion law at the particle/matrix interface based on the extended Mori Tanaka method [14] and cohesive energy equivalence at macro and micro scales. The method linked the macroscopic compaction tension experiment to the microscopic cohesion law at the particle/matrix interface while identifying the critical particle radius to distinguish the hardening and softening behavior of the composites. Chang [15] used the bilinear cohesion model to reflect the mechanical response characteristics of the interface and analyzed the influence law of different interface characteristics on the macroscopic mechanical behavior. Mortazavi [16] promoted the damage model of composite solid propellant under small strain by the interface bonding model and analyzed the damage mechanism of the propellant. Considering the influence of loading rate and temperature on the interface between the propellant and thermal insulation layer, Cui [17] et al. established the Park–Paulino–Roesler (PPR) [18] rate-dependent cohesion zone criterion in order to be more in line with the experimental phenomenon. Ding et al. [19] obtained load–displacement curves at different temperatures and established a model containing polynomial damage variables based on a bilinear cohesion model. The effects of different temperatures on the interface parameters were also analyzed based on the simulated data. Zhao [20] utilized a global cohesive zone model (CZM) to simulate the evolution of composite solid propellants, encompassing debonding, matrix damage, and microcrack propagation, and explored the intrinsic causes of its macromechanical behavior. De Francqueville, F et al. [21] discussed the effect of cohesive zone model (CZM) parameters on the macroscopic mechanical response of non-homogeneous materials. An attempt was made to relate the CZM parameters to the generation and development of localized damage in non-homogeneous materials and to the macroscopic mechanical response of non-homogeneous materials. From the above studies, it can be concluded that the accurate acquisition of propellant mesoscopic structure and cohesion parameters is of great significance in the study of the mechanical response of composite solid propellants.

This study aims to characterize the nonlinear mechanical behavior of solid propellants through meso-mechanical methods. The mechanical shrinkage method was used to generate RVE models with the same size and volume fraction of actual NEPE (Nitrate Ester Plasticized Polyether) propellant particles. A bilinear cohesive unit model was inserted between the propellant particles and the matrix as well as inside the matrix to describe its component properties. By simulating the uniaxial stretching process of NEPE propellants, the effects of interfacial rigidity, interfacial strength, and interfacial failure displacement on the macroscopic mechanical properties of the propellant were investigated. The interface parameters were inverted and analyzed using the Hooke–Jeeves inversion algorithm by combining the experimental and simulation data. The established model and inversion parameters were used to predict the macromechanical behaviors of the propellant at other loading rates to verify their accuracy. Finally, the damage process of the propellant’s mesoscopic interface was analyzed.

## 2. Mesoscopic Model

### 2.1. Representative Volume Element Model

Materials with randomly packed particles are common in nature, such as concrete, composite materials, propellants, etc. NEPE propellants contain a large amount of solid particle fillers, such as aluminum (Al), octogen (HMX), and ammonium perchlorate (AP), and a soft elastomeric binder [22]. Therefore, they should be regarded as nonuniform materials in mesoscopic view [23]. The mechanical properties of NEPE propellants depend on the properties of the matrix, the concentration of components, particle size, and particle distribution. Thus, an appropriate algorithm is needed to construct the RVE model for NEPE propellants. Many researchers have studied random particle packing problems [24,25].

Simulation methods for packing hard particles can be placed into two groups. The first group is referred to as sequential algorithms [26]. Although the algorithm generates particles faster, its volume fraction is not high, which cannot meet the mesoscopic modeling of NEPE propellants with a high volume fraction. The second group, referred to as concurrent algorithms, involves the densification of a fixed number of particles. Molecular dynamics [27], Monte Carlo simulation [28], and mechanical contraction methods [29] are all concurrent algorithms and have been widely used in constructing the RVE model of NEPE propellants.

**Figure 1:**
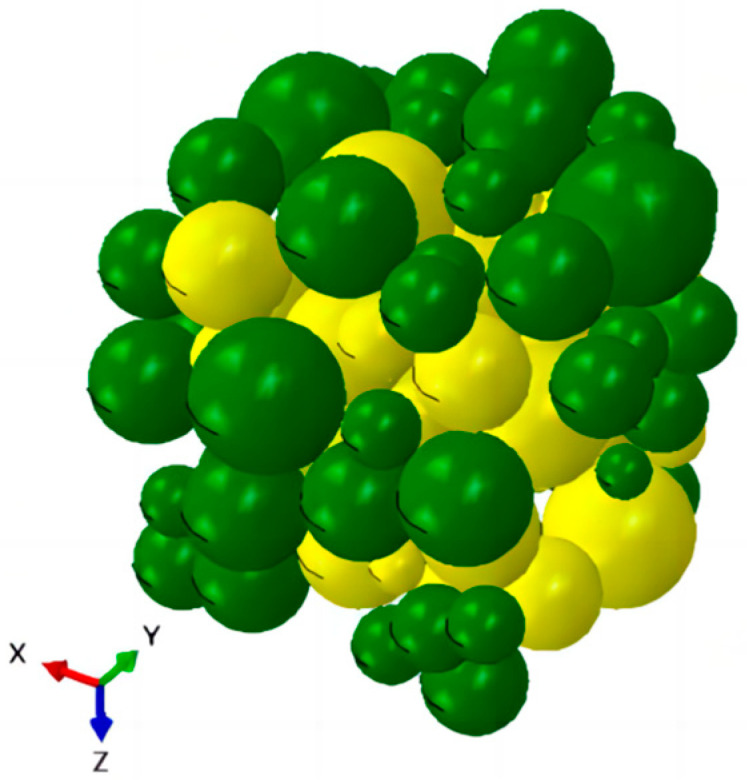
RVE model generated by mechanical compression method (3D).

**Figure 2:**
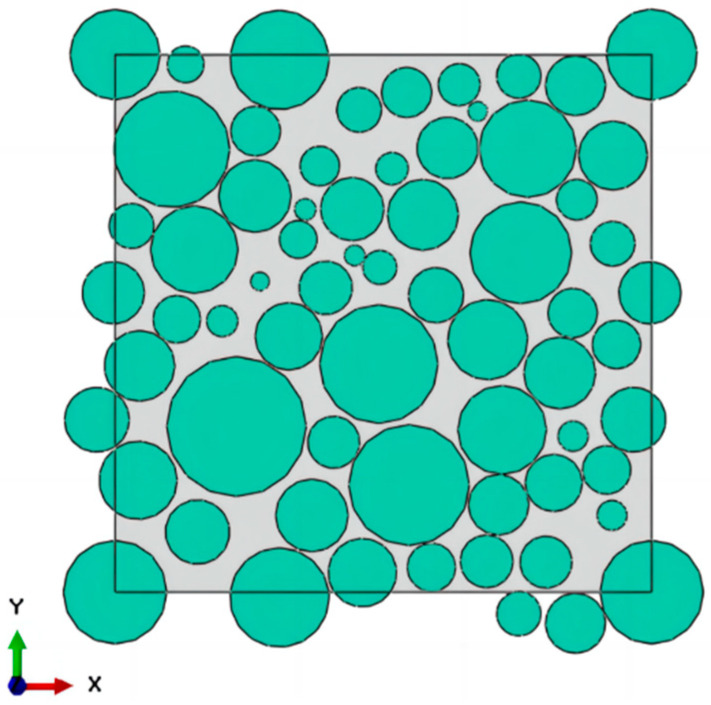
RVE model 2D profile.

In this study, a reference is made to S. R. Williams [29] on the mechanical compression method of randomly filling ellipsoids in a certain space to periodically generate spherical particles as shown in Figure 1 and Figure 2. The particles are considered as isotropic elastic solids and the radii of the particles satisfy a lognormal distribution. The mechanical contraction is based on the idea of a density-quenching system and works as follows.

A unit cube is considered with each side defined as *L_x_*, *L_y_*, and *L_z_*. For convenience of calculation, *L_x_* = *L_y_* = *L_z_*. N points are randomly arranged inside the cube with a normal distribution of radius values. The cube is divided into cells with a certain number of sides, and the number of cells on each side is taken as *n_x_*, *n_y_*, and *n_z_*. The distribution of particles in the cell is calculated, and the movable distance between each particle is also calculated. At the same time, periodic particle generation conditions are given for the particles located around the cube. In the calculation process of the algorithm, the boundary wall of the periodic boundary condition is penetrable, meaning that the particles in the adjacent calculation domain can enter and leave each other, as shown below. The particle radius is iteratively increased proportionally to eliminate the gaps between particles, and the algorithm stops when particles intersect. Finally, an RVE model with a certain volume fraction is generated.

**Figure 3:**
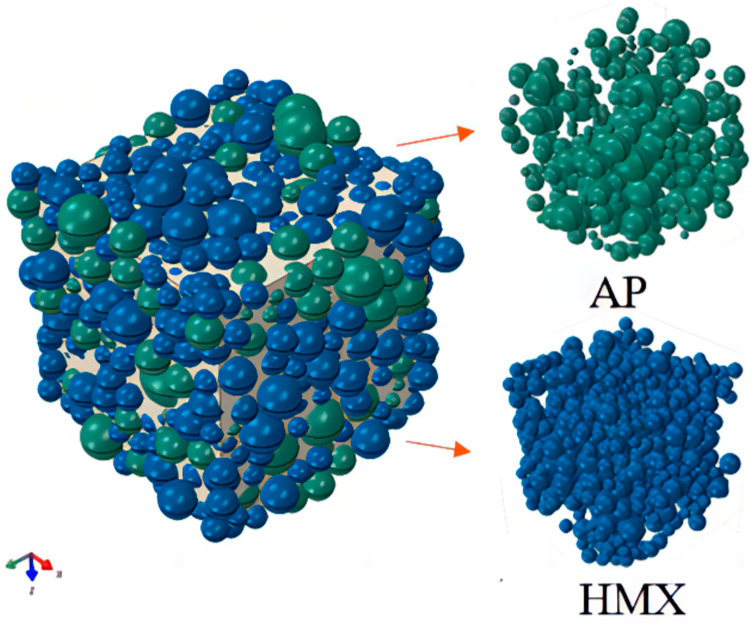
RVE model of the two components.

**Figure 4:**
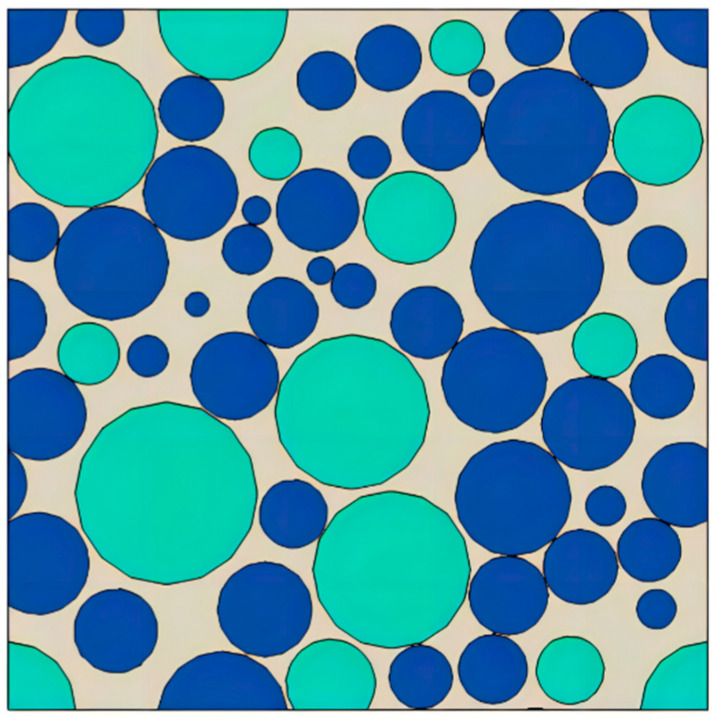
RVE model of NEPE propellant.

The RVE model generator plug-in was used to construct the propellant mesoscopic structure model in ABAQUS software 2020. The RVE model was constructed with reference to the real propellant gradation information to ensure the accuracy of model building. Studies have shown that the RVE size can effectively simulate the meso-mechanical performance response of propellants when the size is 3 to 5 times the maximum particle diameter [30]. The RVE constructed in this study is 1 mm in length, width, and height with components AP and HMX as shown in Figure 3. Due to the increased complexity of computing a 3D RVE, the model chosen for this study is in the 2D plane as shown in Figure 4.

### 2.2. Cohesive Zone Model

In order to study the mesoscopic damage evolution law of NEPE propellant, a mechanical model that can accurately describe the propellant matrix particle interface is required [31].

Since the bilinear cohesion model can well characterize mechanical behaviors such as damage evolution in the cohesive zone under more complex engineering environments, it has been widely used in various studies [32].

**Figure 5:**
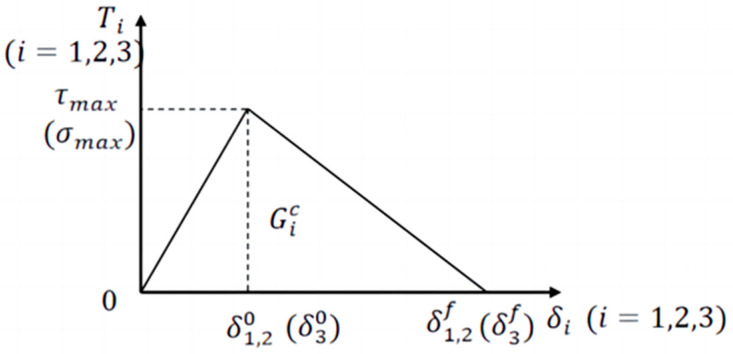
Tension–displacement relations for bilinear cohesion models.

In this study, the disruption of the propellant particle/matrix interface is simulated using zero-thickness cohesive elements that obey the bilinear traction–detachment law [33], as shown in Figure 5.

The intrinsic relationship of the interface is given by the following equation:

(1) $$\left\{\begin{array}{cc} T=K\delta & 0\;<\delta \leq \delta^{0}\\ T=(1+K/\tilde{K})T^{max}-\tilde{K}\delta & \delta^{0}<\delta \leq \delta^{f}\\ T=0 & \delta^{f}<\delta \end{array}\right.$$

where *T^max^* is the interfacial bond strength; *K* and $\tilde{K}$ represent the elastic modulus and softening modulus of the interface, respectively; *δ*^0^ is the interfacial damage critical opening displacement; and *δ^f^* is the interfacial failure opening displacement.

In finite element calculations, the interfacial stress when damage occurs at the interface can be expressed by the following equation:

(2) $$T=\left\{\begin{array}{c} T_{n}\\ T_{t} \end{array}\right\}=\left[\begin{array}{cc} \left(1-D\right)\;K_{nn} & 0\\ 0 & \left(1-D\right)K_{tt} \end{array}\right]\left\{\begin{array}{l} \varepsilon_{n}\\ \varepsilon_{t} \end{array}\right\}$$

where *n*, *t* represent the normal and tangential direction of the interface, respectively; *K_nn_* and *K_tt_* are the elastic stiffness; the interface strain *ε_n_* and *ε_t_* can be derived by the ratio of the interface displacement to the initial thickness of the unit; *D* is the damage variable describing the degree of damage to the interface, *D* = 0 corresponds to the initial interface without damage stage, and *D* = 1 indicates that the interface fails and loses the load-bearing capacity.

The damage variable *D* of the bilinear cohesion model is defined as follows:

(3) $$D=\left\{\begin{array}{cc} 0 & \delta \leq \delta^{0}\\ \frac{\delta^{f}\left(\delta -\delta^{0}\right)}{\delta \left(\delta^{f}-\delta^{0}\right)} & \delta >\delta^{0} \end{array}\right.$$

The damage initiation criterion in the hybrid model is used to determine whether damage occurs at the interface, which is formulated as follows:

(4) $${\left\{\frac{T_{n}}{T_{n}^{max}}\right\}}^{2}+{\left\{\frac{T_{t}}{T_{t}^{max}}\right\}}^{2}=1$$

(5) $${\left\{\frac{G_{n}}{G_{n}^{c}}\right\}}^{2}+{\left\{\frac{G_{t}}{G_{t}^{c}}\right\}}^{2}=1$$

where *G_c_* is the interfacial bonding energy and *G_n_* and *G_t_* are the normal and tangential interfacial energy release rates, respectively, and generally have *G_n_* = *G_t_*.

### 2.3. Numerical Computational Model of NEPE Propellants

1. NEPE propellant mesoscopic modeling

**Figure 6:**
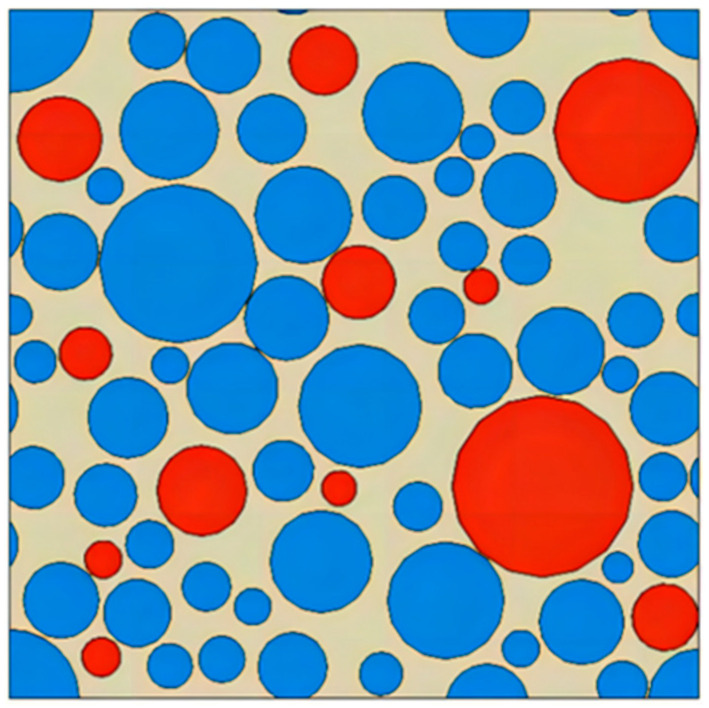
RVE model with a volume fraction of 0.6. (AP in red and HMX in blue).

Based on the grading information of NEPE propellant formulation, as shown in Table 1, the propellant oxidizer particles were simplified to a spherical shape and combined with the characteristics of random distribution of particles in the matrix; the RVE model of NEPE propellant was generated using mechanical compression method. The propellant RVE model was constructed with a solids content of 0.6 and a 1:3 volume ratio of AP to HMX particles as shown in Figure 6. The size of the particles in the computational domain is uniformly distributed, as shown in Figure 6. The smaller particles are more abundant and are distributed among the larger particles, filling the pores between large particles.

Cohesive grid cells were inserted both at the particle/matrix interface and inside the matrix.

2. Material parameters

**Figure 7:**
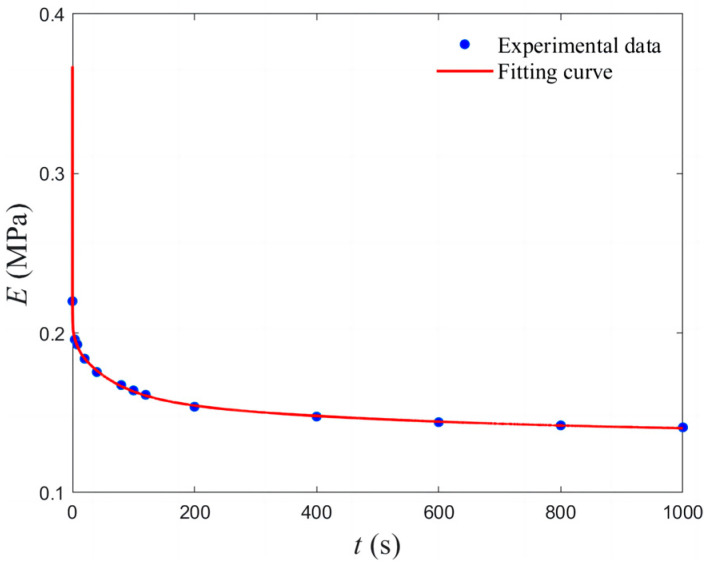
NEPE propellant matrix stress–relaxation curves.

The material parameters of the NEPE propellant compositions are listed in Table 2. Stress–relaxation tests were performed on the NEPE propellant matrix specimens to obtain valid test results and average them. The stress–relaxation test curves of the propellant matrix are fitted in the form of a Prony series, and the fitted expression is given as

(6) $$E(t)=E_{\infty }+\sum_{i=1}^{n}E_{i}\exp\left(\frac{t}{\tau_{i}}\right)$$

where *E*_∞_ is the equilibrium modulus, *E_i_* and *τ_i_* are the modulus and relaxation time of the *i*th Maxwell cell, respectively, and *t* is the time. The fitting results are shown in Figure 7, from which it can be seen that the fitting results are in good agreement with the experimental results. The specific parameters of the relaxation modulus of the NEPE propellant matrix are shown in Table 3.

3. Boundary conditions

**Figure 8:**
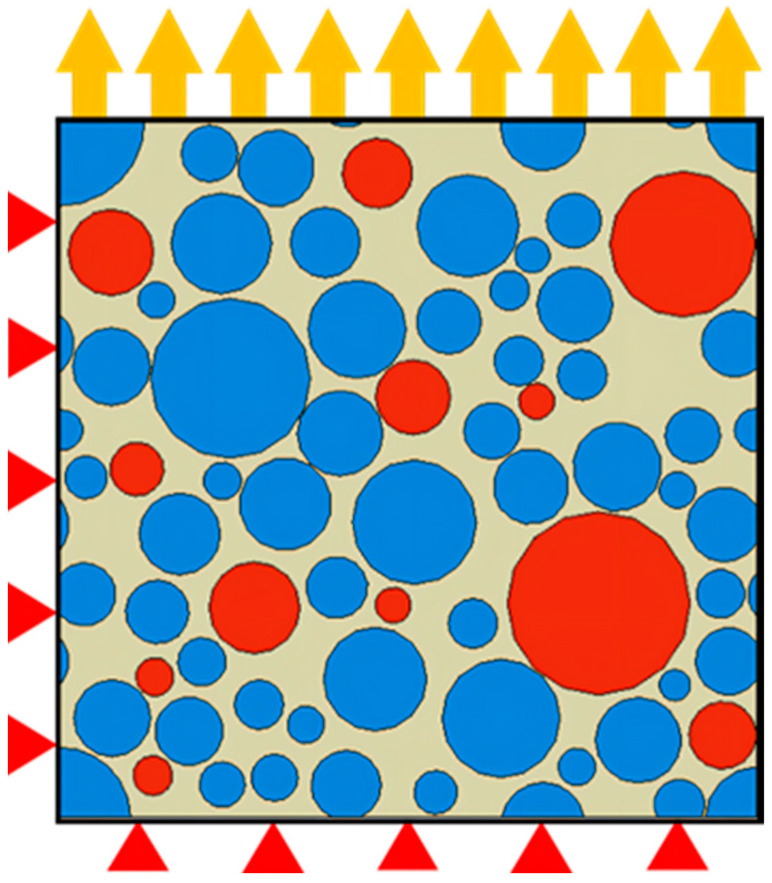
Boundary conditions of the RVE model.

The application of periodic boundary conditions often relies on the division of periodic grids. Because the mesoscopic structure of NEPE propellant is very complex and is not suitable for drawing periodic grids, some studies have shown that uniform boundary conditions can be used in the simulation calculation of mesoscopic structures instead of periodic conditions. The NEPE propellant RVE model and boundary conditions imposed are shown in Figure 8.

4. Grid-independent verification

**Figure 9:**
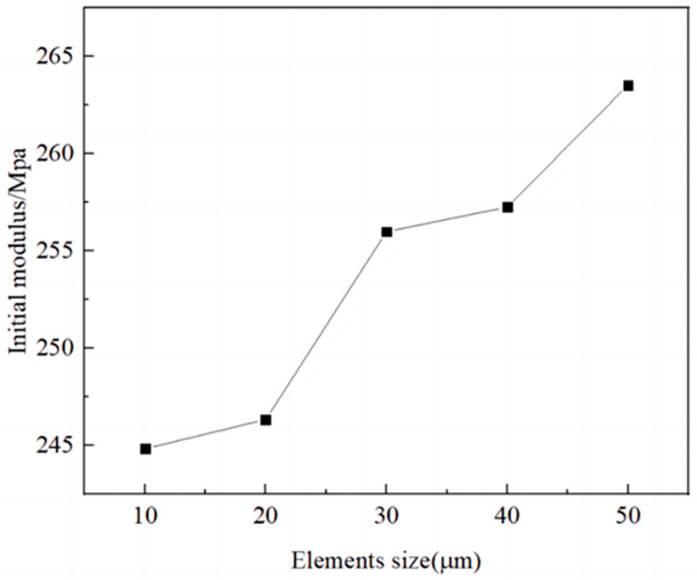
Grid-independent verification.

**Figure 10:**
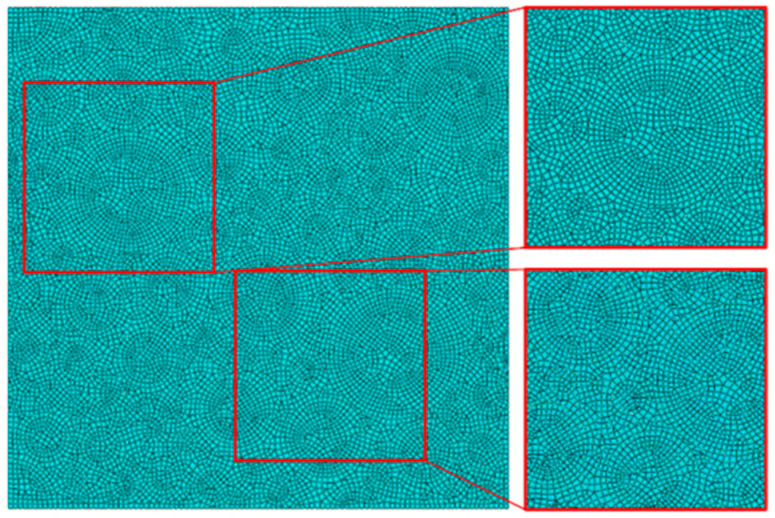
Grid division.

For numerical calculations, grid-independent verification is mandatory. The mesh sizes of the RVE model were divided into 10 μm, 20 μm, 30 μm, 40 μm, and 50 μm. The initial modulus of each mesh size was calculated separately to investigate the mesh independence of the model, and the final result is shown in Figure 9. It can be seen that with a reduction in the model mesh size, the initial modulus of the propellant gradually decreases. The final grid size was chosen to be 10 μm, which is more accurate. The grid division is shown in Figure 10. The number of grids for each component is shown in Table 4.

## 3. Numerical Calculations and Inversion of Interface Parameters

### 3.1. Effects of Interface Parameters

**Figure 11:**
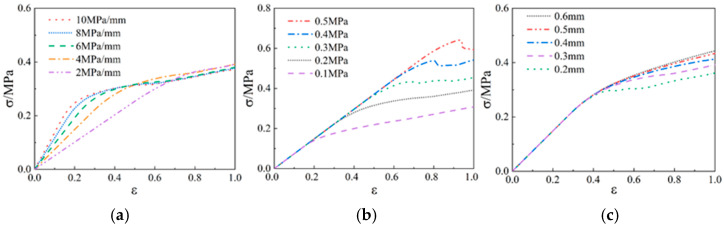
Three parameters influence the response of the mechanical curve: (**a**) interfacial rigidity; (**b**) interfacial strength; (**c**) interfacial failure displacement.

A bilinear cohesive zone model was used to characterize the interface damage propagation. This cohesion law has three independent parameters: interfacial rigidity, interfacial strength, and interfacial failure displacement. The effects of the three parameters in the bilinear cohesive zone model on the stress–strain behavior of the NEPE propellant were analyzed. The results are shown in Figure 11.

The study of different interface parameters shows that the three parameters of the interface have a significant effect on the mechanical properties of the propellant. By changing the value of the interfacial rigidity in the mesoscopic model, the stress–strain curve of the propellant is calculated as shown in Figure 11a. From the figure, it can be seen that the interfacial rigidity mainly affects the slope of the linear section of the curve. The larger the interfacial rigidity is, the larger the slope of the curve is. In the same way as the study of interfacial rigidity, by changing the value of interfacial strength in the mesoscopic model, the propellant stress–strain curve is calculated as shown in Figure 11b. From the figure, it can be seen that the interface strength controls the initiation and evolution process of propellant damage. The linear segments of the curves corresponding to different interfacial strengths basically overlap. The biggest difference between the curves is that the larger the interfacial strength is, the longer the curve rises to the peak stress, and its interfacial stress finally reaches the value of interfacial strength. The greater the interfacial strength, the higher the elastic energy stored in the interface, and the interface damage releases a large amount of stress, resulting in a significant reduction in propellant stress. Continuing to stretch until the interface fails, the interface no longer carries and transmits the load, the matrix is elongated under the load, and the curve exhibits the mechanical properties of the matrix with a slow increase in stress. Similarly, by changing the value of the interfacial failure displacement in the mesoscopic model, the propellant stress–strain curve is calculated as shown in Figure 11c. It can be seen from the figure that the linear segments of all the curves basically coincide. This is because when the other two variables are certain, the interface damage of the mesoscopic model under the same strain is generated almost simultaneously. The interfacial damage displacement has a significant effect on the large deformation stage of the matrix after the propellant interface is dehumidified. It can be obtained by comparative analysis. The above study is basically consistent with the effect of interfacial parameters of composite solid propellants on their mechanical properties in the literature [34].

### 3.2. Inversion of Interface Parameters

Currently, the mesoscopic interfacial parameters are mostly obtained empirically, with a large error. In this study, an inversion algorithm was used to obtain the final interface parameters.

**Figure 12:**
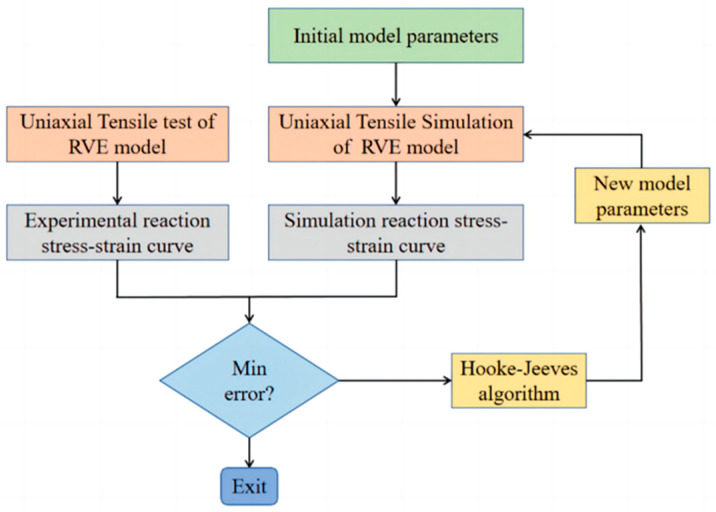
Flow chart of the inversion analysis.

**Figure 13:**
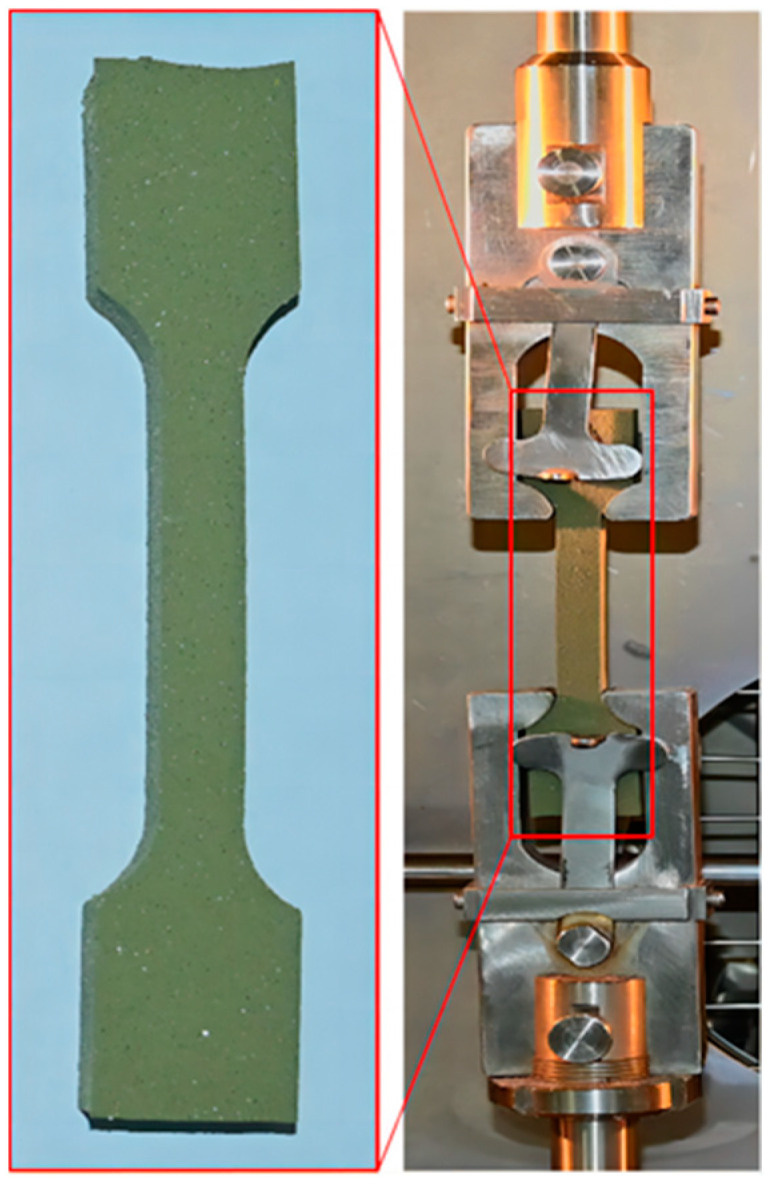
Propellant specimen and uniaxial stretching process.

**Figure 14:**
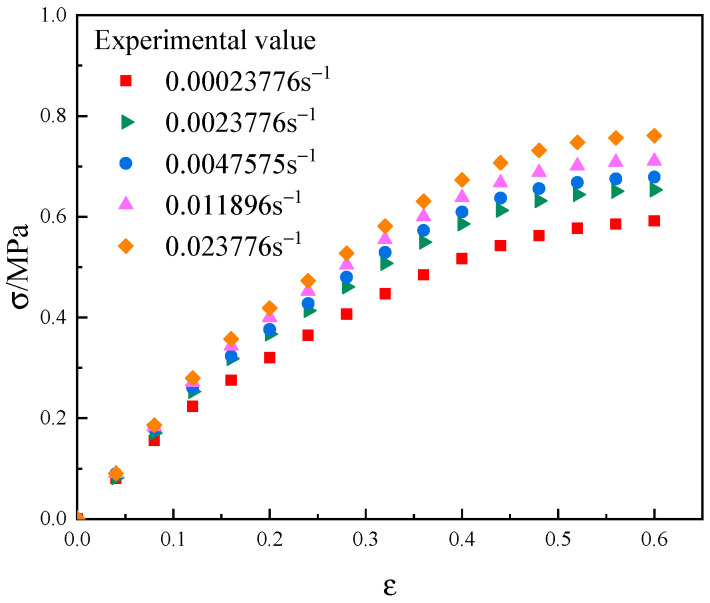
Stress–strain curves of NEPE propellant at different strain rates.

The inversion algorithm involves three parts: the Hooke–Jeeves algorithm [35], the invocation of ABAQUS, and the construction of the objective function. The Hooke–Jeeves algorithm is the main program of the inversion analysis, which is calculated by adjusting the parameters of the object, invoking the ABAQUS software, and then comparing the calculated values with the experimental values to compute the value of the objective function. The final optimization goal is achieved through cyclic calculation. The inversion flow chart of NEPE propellant interface parameters is shown in Figure 12. The inversion algorithm requires actual test curves, so uniaxial tensile tests were performed on the NEPE propellant. The tensile specimen of the NEPE propellant was made into a standard dumbbell specimen and tested in accordance with the “Unidirectional Tensile Test Method for Composite Solid Propellants” QJ924-85 [36] as shown in Figure 13. Room temperature conditions were maintained, the tensile speeds were set to be 1 mm/min, 10 mm/min, 20 mm/min, 50 mm/min, and 100 mm/min for the uniaxial tensile test, and the corresponding strain rates were 0.00023776 s^−1^, 0.0023776 s^−1^, 0.0047575 s^−1^, 0.011896 s^−1^, and 0.023776 s^−1^. The stress–strain test curves of the NEPE propellant with different strain rates are shown in Figure 14.

**Figure 15:**
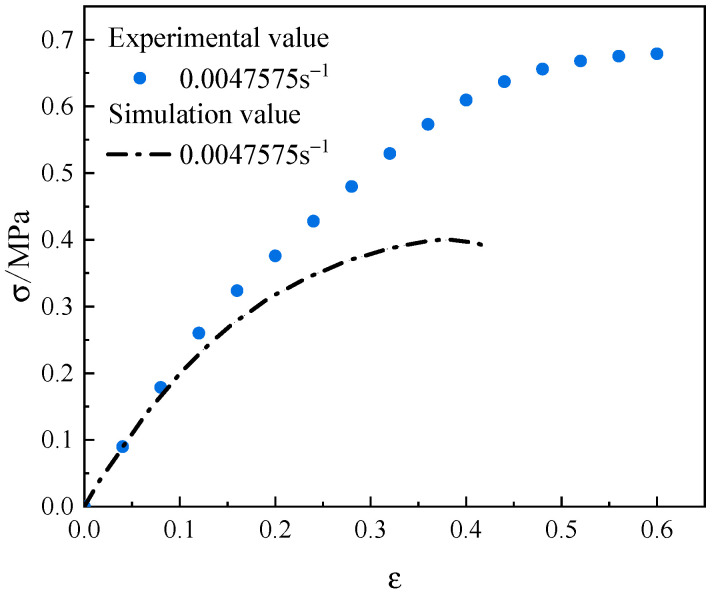
NEPE propellant numerical calculation results.

Taking the RVE model with a solid content of 0.6 as an example, the material parameters were set, and the interface parameters at the particle/matrix interface and inside the matrix were given initial values based on empirical data. An axial unique load was applied to the RVE model to obtain the stress–strain curves of the process, as shown in Figure 15. From Figure 15, it can be seen that the simulation curves obtained based on the initial interface parameters are quite different from the actual test curves. Therefore, it is necessary to optimize the inversion of the interface parameters to improve the consistency between the simulation curves and the actual experimental curves.

**Figure 16:**
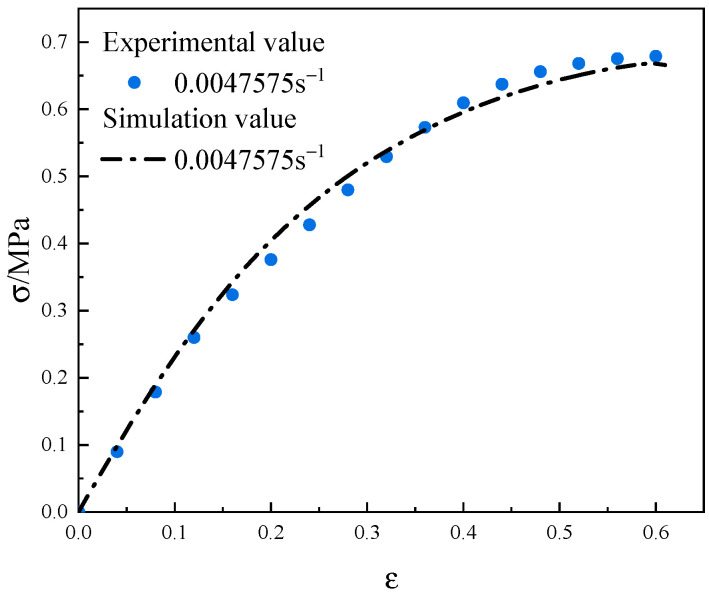
Comparison of inversion simulation results with experimental results.

Combined with the uniaxial tensile curve with a strain rate of 0.0047575 s^−1^ and the inversion analysis, the interface parameters at the particle/matrix interface as well as inside the matrix were obtained by finite element calculations. The simulation results are shown in Figure 16, and the inversion-optimized interface parameters are shown in Table 5.

From Figure 16, it is evident that after the inversion analysis, the simulation curves and test curves are in good agreement.

### 3.3. Interface Parameter Validation

**Figure 17:**
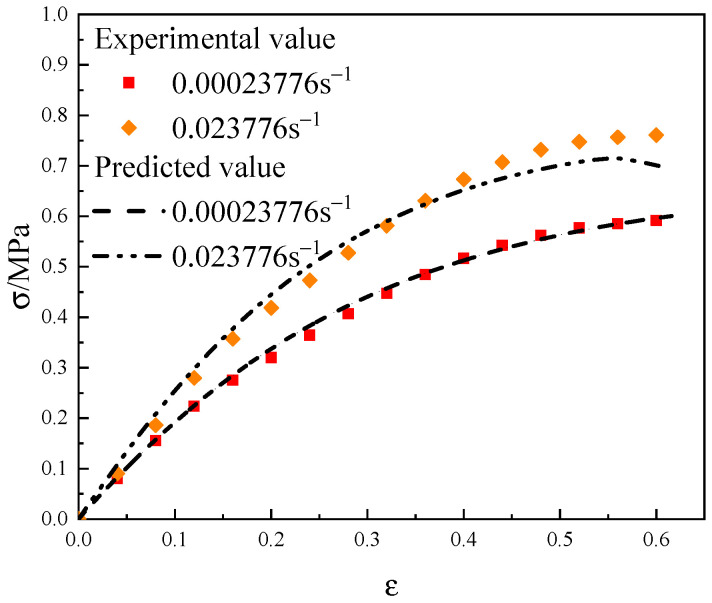
Parameter verification.

Under the same model and the same interface parameters, the macroscopic stress–strain response of NEPE propellants at other strain rates can be predicted, and the accuracy of the model and the interface parameters can be verified by the test curves and the prediction curves. Uniaxial tensile tests with strain rates of 0.00023776 s^−1^ and 0.023776 s^−1^ were used for the validation tests. A comparison of the predicted curves with the test curves is shown in Figure 17. From Figure 17, it can be seen that the basic features and trends of the predicted curves and the test curves are relatively consistent, indicating that the established mesoscopic numerical model can effectively reflect the adhesion between the interface between the particles and the matrix in the actual NEPE propellant and its macroscopic mechanical behavior.

## 4. The Damage Fracture Evolution of the Propellant

**Figure 18:**
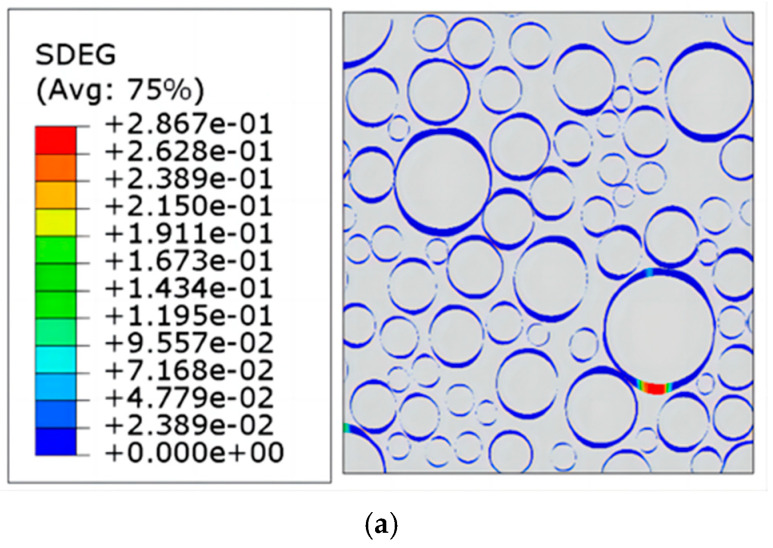

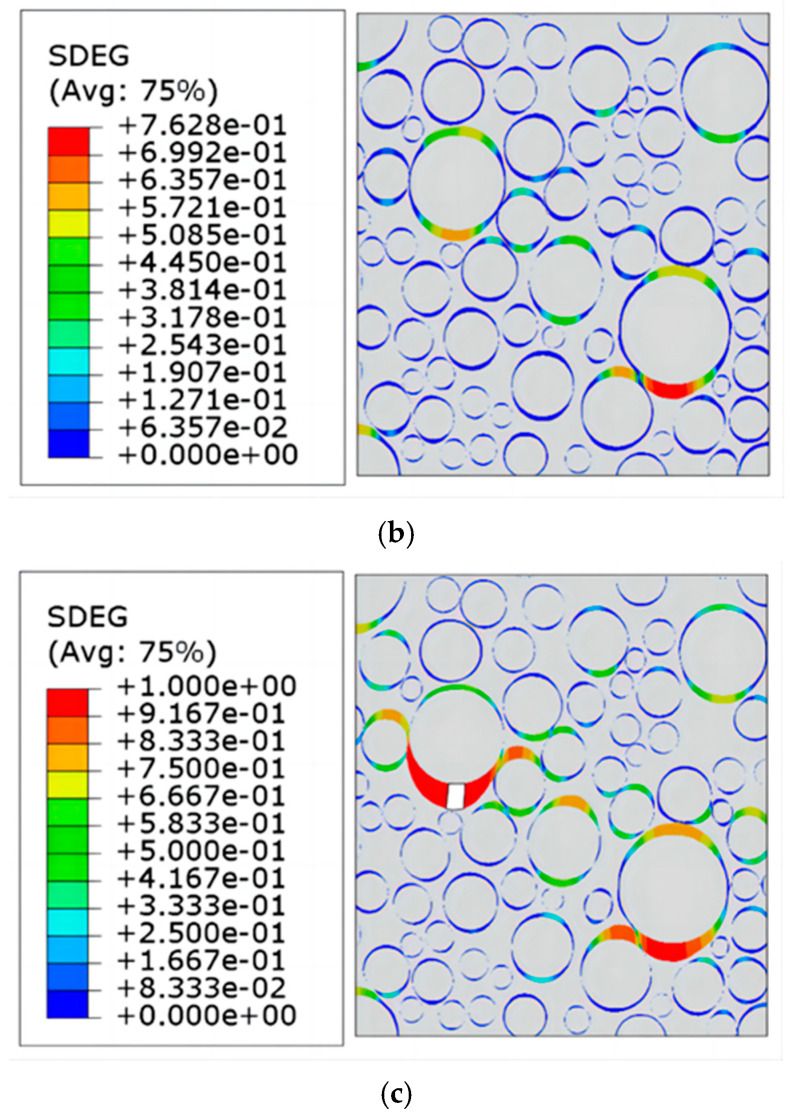
Stiffness decay rate plots for mesoscopic damage evolution of NEPE propellant. (**a**) ε = 13.25%; (**b**) ε = 40.70%; (**c**) ε = 61.67%.

**Figure 19:**
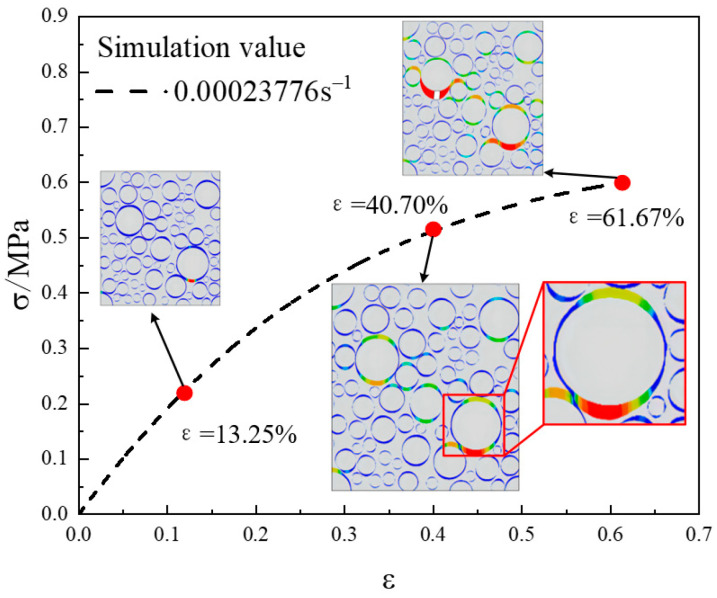
Simulated stress–strain curve at 0.00023776 s^−1^ strain rate.

Tensile simulations with a strain rate of 0.00023776 s^−1^ were performed for a 2D RVE model with a solid content of 0.6. The variation in the stiffness decay rate plots of the NEPE propellant mesoscopic structure at strains of 13.25%, 40.70%, and 61.67% is shown in Figure 18. The locations of the three strains are indicated in the simulated stress–strain curves in Figure 19.

Through the SDEG distribution cloud diagram, the evolution mechanism of propellant mesoscopic damage and destruction behavior can be revealed from the mesoscopic level. When the overall strain is small, interfacial debonding forms at the nearby edges of the larger particles. This is due to the different nature of the propellant component materials, resulting in an uneven distribution of stress–strain within the propellant. The modulus of the particles is much larger relative to the matrix, and thus, the matrix deforms more while the particles deform less during the stretching process. This makes the interface between the matrix and the particles a weak position, and interfacial debonding occurs when the overall strain is small. When the overall strain continues to increase, the debonding at the interface develops further, and more and more micropores are formed. In the vicinity of large particles, the local strain of the matrix is higher, especially at locations where the particles are more concentrated. Under loading, the debonding at the interface at these locations becomes more pronounced. When the overall strain increases further, the micropores produced by the propellant interfacial debonding will appear as obvious cracks. As the load continues to increase, the separation of the particles from the matrix widens. Gradually, these micropores created by interfacial debonding begin to converge, eventually causing the matrix to tear, resulting in cracks and hence macroscopic damage to the propellant. This is in agreement with the findings in the literature [37].

## 5. Conclusions

1. The NEPE composite solid propellant mesoscopic model established based on the mechanical contraction method and bilinear cohesion unit can effectively reflect the mesoscopic characteristics of the propellant.
2. The constructed mesoscopic simulation model can effectively reflect the strain rate correlation of the mechanical properties of NEPE composite solid propellants. The macroscopic stress–strain relationship of NEPE propellants can be effectively predicted in the range of 0.00023776 s^−1^~0.023776 s^−1^ strain rate.
3. The damage mechanism of NEPE propellants under external loading can be obtained by analyzing the evolution of NEPE propellant mesoscopic damage. At the early stage of loading, the strain is small, there is no damage in the mesoscopic interior, and the stress–strain curve is linear. When continuing loading, damage occurs at some positions. With further loading, the damage at the interface between the particles and the substrate increases, with some interfacial failures and microcracks appearing. The matrix cracks further and the propellant fractures and fails.

## Figures and Tables

**Table 1:** Formulation grading information.

Components	AP	HMX
Average size (μm)	120	75
Interval size (μm)	50~190	25~130

**Table 2:** Mechanical parameters of NEPE propellant components.

Components	Young’s Modulus (MPa)	Poisson’s Ratio	Density (kg/m^3^)
AP	32,450	0.130	1950
HMX	29,500	0.300	1900
Matrix	*E*(*t*)	0.495	1200

**Table 3:** Prony series.

*i*	∞	1	2	3	4	5	6
*E_i_*/MPa	0.3679	0.0788	0.0645	0.0205	0.0100	0.0330	0.0238
*τ_i_*/s	–	0.0041	0.0231	0.3948	6.1445	64.6894	542.4027

**Table 4:** Number of grids for each component.

Components	Number of Grids
AP particle	1905
HMX particle	5456
Matrix	5645
Particle/matrix interface bonding unit	1553
Matrix neighboring unit interface bonding unit	10,443

**Table 5:** Particle/matrix interface and matrix cohesion unit parameters.

Components	σ^max^/MPa	K/(MPa/mm)	*δ^f^*/mm
Particle/matrix interface	0.50	400	2.0
Matrix	0.65	300	2.5

## Data Availability

Data are contained within the article.
